# Hybrid biocomposites from polypropylene, sustainable biocarbon and graphene nanoplatelets

**DOI:** 10.1038/s41598-020-66855-4

**Published:** 2020-07-01

**Authors:** Ethan Watt, Mohamed A. Abdelwahab, Michael R. Snowdon, Amar K. Mohanty, Hamdy Khalil, Manjusri Misra

**Affiliations:** 1 Bioproducts Discovery and Development Centre, Department of Plant Agriculture, Crop Science Building, University of Guelph, Guelph, Ontario N1G 2W1 Canada; 2 School of Engineering, Thornbrough Building, University of Guelph, Guelph, Ontario N1G 2W1 Canada; 3 Woodbridge Foam Corporation, 4240 Sherwoodtowne Boulevard, Mississauga, L4Z 2G6 Ontario Canada

**Keywords:** Nanoscale materials, Materials chemistry, Polymer chemistry

## Abstract

Polypropylene (PP) is an attractive polymer for use in automotive parts due to its ease of processing, hydrophobic nature, chemical resistance and low density. The global shift towards eliminating non-renewable resource consumption has promoted research of sustainable biocarbon (BioC) filler in a PP matrix, but this material often leads to reduction in composite strength and requires additional fillers. Graphene nano-platelets (GnPs) have been the subject of considerable research as a nanofiller due to their strength, while maleic anhydride grafted polypropylene (MA-g-PP) is a commonly used compatibilizer for improvement of interfacial adhesion in composites. This study compared the thermo-mechanical properties of PP/BioC/MA-g-PP/GnP composites with varying wt.% of GnP. Morphological analysis revealed uniform dispersion of BioC, while significant agglomeration of GnPs limited their even dispersion throughout the PP matrix. In the optimal blend of 3 wt.% GnP and 17 wt.% BioC biocontent, tensile strength and modulus increased by ~19% and ~22% respectively, as compared to 20 wt.% BioC biocomposites. Thermal stability and performance enhancement occurred through incorporation of the fillers. Thus, hybridization of fillers in the compatibilized matrix presents a promising route to the enhancement of material properties, while reducing petroleum-based products through use of sustainable BioC filler in composite structures.

## Introduction

Plastics have been embraced in the automotive industry specifically due to their durability, ease of moulding and low density, with percent plastic by weight in automobiles rising 75% from roughly 200 kg per car in 2014 to 350 kg in 2020 according to a study by IHS chemical^1^. Polypropylene (PP) is one such plastic that is commonly used in exterior and interior automotive parts such as bumpers, dashboards and hoods. PP sees wide use due to its high heat and chemical resistance, while still maintaining strong mechanical properties and a low density of 0.95 g/cm^3^ at a comparatively low cost^2^.

Increased dependence on plastics has raised concerns about petroleum usage and waste disposal. Plastics are largely non-biodegradable, making disposal at landfills problematic, while incineration releases toxins that threaten the environment and contributes to climate change^3^. In 2018, PP was the most demanded plastic, accountable for 19.3% of global plastic production as found in the 2019 PlasticsEurope Market Research Group consensus, meaning a reduction in the use of this polymer could significantly reduce oil dependence^4^. Thus, the incorporation of biobased fillers into a PP matrix has been a growing avenue of research. However, integration of biobased fillers presents several key challenges, including maintaining desirable thermal and mechanical properties at low weight and cost.

Biocarbon (BioC) is one such biobased material, produced through a process known as pyrolysis. Pyrolysis is the heating of low energy organic materials in the absence of oxygen, resulting in the production of higher energy syngas, bio-oil and BioC^5^. These are produced in varying ratios, depending on pyrolysis temperature, residence time and type of organic material^5^. BioC already sees use as a soil amendment and in wastewater treatment, but higher volume applications are required to increase the feasibility of pyrolysis as BioC is typically produced in excess^6^. The physical structure and properties of BioC vary widely depending on pyrolysis conditions. Heating up to 900 °C results in the release of volatiles, eliminating functionality and subsequently increasing surface area through greater material porosity^7^. Meanwhile, pyrolysis at 500 °C has produced BioC consisting of a 3D network of benzene rings with a high amount of functional groups in its structure, leading to the material having a more polar nature^8^.

Use of BioC for automotive parts presents several key advantages, most prominent being the introduction of biocontent through reduction of the PP matrix. Furthermore, BioC can be generated from a wide range of waste feedstocks or undervalued biomass that would otherwise be discarded, leading to reduced production costs compared to other carbon-based fillers and also contributing to a circular economy^9^. The density of BioC typically ranges from 1.3–1.4 g/cm^3^ depending on pyrolysis temperature and feedstock, which is much less than traditional mineral fillers like talc, described with a density between 2.6–2.7 g/cm^3^ ^6^. This is advantageous, as lower density composite parts lead to direct cost-savings through improvement of fuel economy in automobiles. As a result, it has been found that BioC serves as an attractive low-cost support for nanoparticle incorporation in composites^10^. In a recent study comparing pyrolysis temperature, Behazin *et al*.^11^ found that nonpolar 900 °C BioC had better compatibility with an ethylene-octene copolymer modified PP matrix as compared to 500 °C BioC due to the loss of functionality at high pyrolysis temperatures. They described that this should increase the affinity of BioC with the non-polar polymeric chains of the matrix, but found that, overall, both temperatures led to a decrease in mechanical strength. However, pyrolysis does lead to a more thermally stable structure, which is attractive for high-temperature applications^12^. Despite poor compatibility, pyrolysis conducted at low temperatures produces a higher yield of BioC, making it more feasible for industrial use^13^. The effect of BioC particle size has also been analyzed, with smaller particles corresponding to superior mechanical properties and dispersion, indicating the need for ball milling following pyrolysis^14,15^.

Commonly, functionalized PP is added to the interface between polar fillers, such as low temperature BioC, and the non-polar polymeric chains of PP to improve adhesion. The amount added is of great importance, as excess compatibilizer will saturate the matrix, ceasing any further participation in improvement of interfacial adhesion and leading to raised costs^16^. Typically, optimal compatibilizer concentrations range from 1–5 wt.% depending on the composite material in question. Studies have found maleic anhydride grafted polypropylene (MA-g-PP) to be a good compatibilizer between PP and BioC^17,18^. MA-g-PP results in separate dispersion of phases between BioC and PP, improving both stiffness and toughness of composites.

The use of nanomaterials is a promising new avenue of research to further improve mechanical and thermal properties of composites. Graphene is one such nanomaterial, with a two-dimensional hexagonal lattice structure that gives it incredible stability and strength^19^. Graphene is simply a single atomic layer of graphite, and multiple derivatives of graphene exist, including graphene nanoplatelets (GnPs), graphene oxide and reduced graphene^20^. Of these, GnPs hold great promise as a reinforcing agent in composites due to their unique morphology, lower cost and low density^21^. GnPs are comprised of small planar stacks of graphene sheets in the shape of platelets, formed from chemical or liquid phase exfoliation of bulk graphite^22^. This forms a mix between the structure of graphite and graphene, as it consists of single and few-layer graphene (2–10 layers), intermixed with nanostructured graphite. Their classifications depend on the thickness, lateral size, and elemental concentration. In any given batch, thickness can vary from 0.34 nm to 100 nm and the number of graphene layers is typically between 10–60^23,24^. This structure leads to the high surface area and stability of GnPs, and research has found that their integration into composites can increase mechanical, thermal and electrical conductivity properties^25^. GnPs are also less expensive than many common nano-fillers, such as carbon nanotubes or graphene oxide, and can bring performance improvements at low loadings to offset this cost^26^. GnPs lack functionalization, desirable for a non-polar PP matrix as compared to reduced graphene oxide, and have a low density for their comparably high surface area as compared to other nanofillers, like nanoclay. Jun *et al*.^25^ analyzed the effect of varying GnP size on a PP matrix and found that particle diameters below 15 µm exhibited a higher level of GnP dispersion in the matrix, improving stress transfer and hindering crack propagation. Larger particle size led to agglomeration that hindered improvement of mechanical and thermal properties^25^. Homogenous dispersion of this nanomaterial can prove difficult, as strong *π- π* interactions and *van der Waals* forces between graphene sheets increase the tendency to agglomerate^27^. Interestingly, the addition of both BioC and GnPs into a PP matrix has not been reported in literature and indicates the need for further study.

BioC can improve thermal properties of composites while simultaneously reducing petroleum usage. Its immiscibility with a PP matrix has been shown to be greatly reduced through the addition of MA-g-PP, while incorporation of GnPs has been seen to enhance both mechanical and thermal properties in many composites^25,28^. It is proposed that the combination of BioC and GnPs in compatibilized PP blends has the potential to enhance properties while simultaneously increasing biocontent and maintaining a low-density material. The aim of this research is to study the influence of GnP addition on performance of compatibilized PP/BioC composites. Mechanical, thermal and morphological characterizations of these composites were performed to determine the feasibility of their use in automotive applications.

## Results and discussion

### Characterization of powder samples

A comparison of particle size and aspect ratio of the BioC and GnP powder used was conducted through SEM imaging, and is outlined in Table 1. As many GnPs were embedded in the injection flow direction within the PP matrix, their average measured particle size was lower than 5 µm. This was due to only platelet edges being primarily visible when viewing samples in the SEM in the perpendicular direction to the flow. The measured particle size of BioC particles following 2 hours of ball milling matched well with a recent study^14^. Fourier transform infrared spectroscopy was also conducted on these powders and soyhull meal to further characterize their functionality, with the spectra displayed in Fig. S1. FTIR of raw soyhull meal and BioC agreed with a previous paper (Fig. S1a,b)^7^. However, FTIR of GnP showed an absence of any functional groups, as shown in Fig. S1c.

**Table 1:** Measured characteristics of the BioC and GnP powders used. Particle size and aspect ratio were measured through use of SEM. Data in bold represents the range which holds the maximum particle population.

Powder Sample	Particle size distribution (%)						Average particle size (µm)	Aspect Ratio	Surface Area (m^2^/g)
	0–0.5 µm	0.5–1 µm	1–3 µm	3–5 µm	5–8 µm	8–10 µm			
BioC	0.22	12.59	**69.44**	13.92	3.61	0.22	2.07	0.65	10.7
M5 GnP	**47.23**	32.95	16.22	2.83	0.77	/	0.78	0.70	120–150

**Figure 1:**
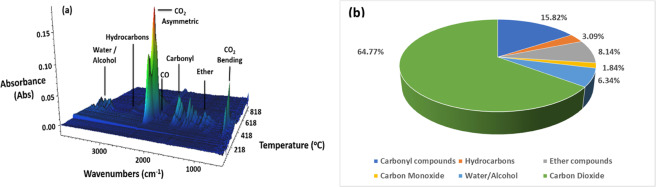
(**a**) 3D surface plot of soyhull fiber generated by TGA-FTIR analysis for evolved gas products during heating and **(b)** relative amounts of volatiles generated.

TGA-FTIR analysis was performed on the raw soyhull feedstock to examine the volatiles released during the pyrolysis process, as observed in Fig. 1a. The major volatile shifts identified were water/alcohol at ~3600 cm^−1^, hydrocarbons at ~2950 cm^−1^, carbon monoxide at ~2150 cm^−1^, carbonyl compounds at ~1750cm^−1^, ether compounds at ~1200 cm^−1^, and carbon dioxide asymmetric and bending stretches at ~2350 cm^−1^ and ~650 cm^−1^, respectively. As identified by the absorbance peaks and Fig. 1b, the major constituent of the volatiles released consisted of carbon dioxide, with minor contributions from carbonyl, ether, and alcohol compounds. The Gram-Schmidt curve of soyhull fiber can additionally be seen in Fig. S2.

### Composite morphology

**Figure 2:**
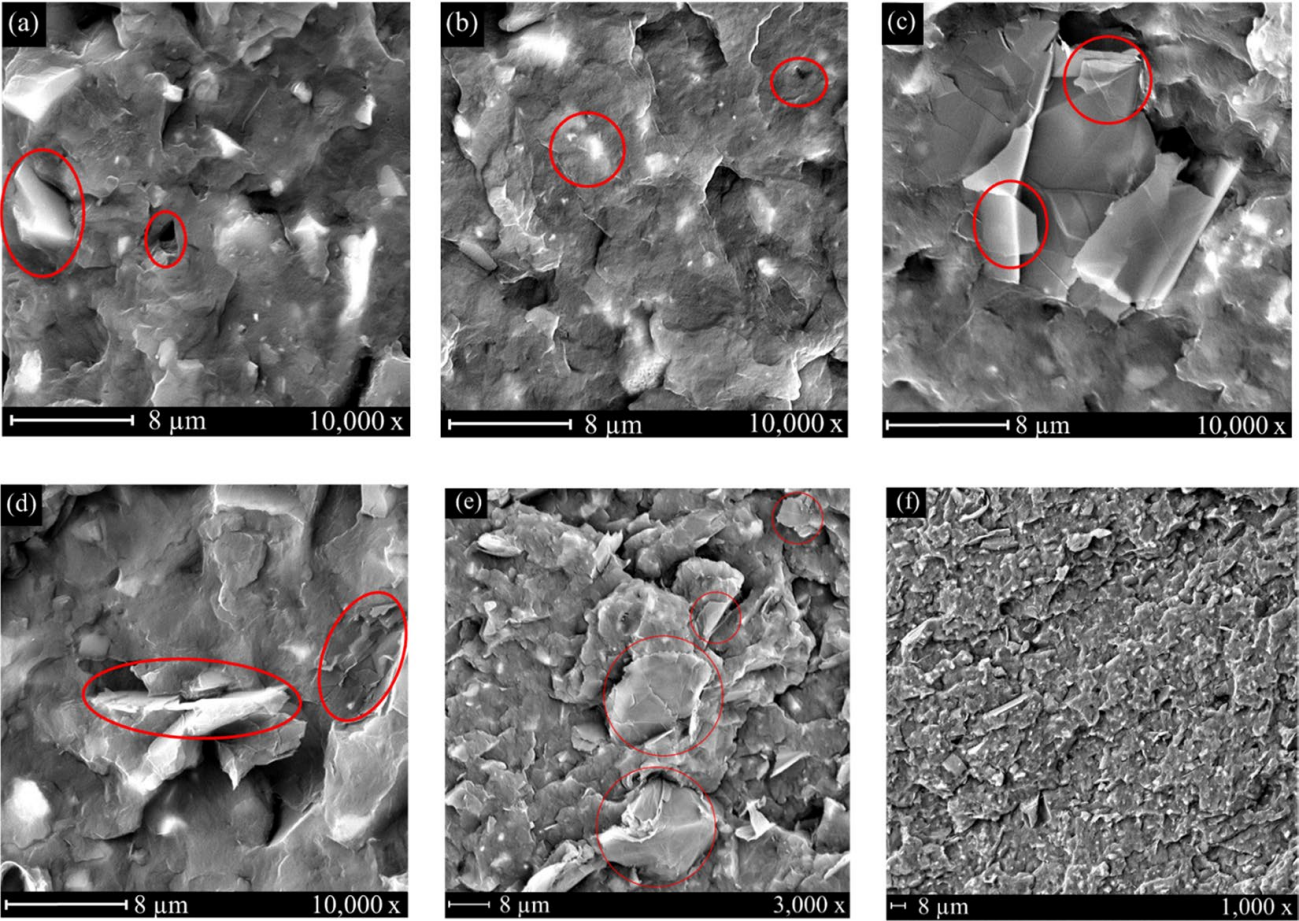
SEM micrographs of cryo-fractured composites for **(a)** the uncompatibilized blend (80/20/0/0) and **(b)** in the presence of compatibilizer (77/20/3/0), with BioC/matrix interactions circled. Agglomerated GnPs were identified in both **(c)** 1 wt.% composites (77/19/3/1) and **(d)** 3 wt.% composites (77/17/3/3). Excessive GnP agglomeration was identified in 5 wt.% composites at 3,000x magnification in **(e)**, and a low 1,000x magnification cross-section of the fracture surface for 3 wt.% GnP loading is visible in **(f)**.

Figure 2 shows SEM images of impact bars of ~3.3 mm thickness that were cryo-fractured to expose the internal structure for high resolution imaging. Incorporation of 20 wt.% BioC into a PP matrix is shown in Fig. 2a. Phase separation was identified and circled between the PP matrix and the BioC particles, with significant pull-outs after fracture. This was due to a lack of interaction between the non-polar PP chains and functionalized BioC. Addition of MA-g-PP compatibilizer significantly improved adhesion, as particles of BioC were embedded in the matrix and there was less evidence of pull-outs occurring, as shown in Fig. 2b. Overall, the fracture surface was significantly smoother with addition of compatibilizer and consequently led to increased interaction between BioC particles and the matrix. The sample after addition of 1 wt.% GnP to compatibilized PP/BioC composites is shown in Fig. 2c, where significant agglomeration was identified. As the thickness of a GnP is between 6–8 nm, the agglomeration perpendicular to the matrix consisted of a multitude of GnP stacks, with two transparent individual layers identified. Uniform dispersion was lacking for GnPs beyond the agglomerated stack, due to strong intermolecular forces present between the sheets. GnP loading at 3 wt.% indicated significant agglomeration, with stacks identified both parallel and perpendicular to the PP matrix, as shown in Fig. 2d. Along with intermolecular forces, agglomeration was increased due to residual moisture content in the material. Excessive GnP agglomeration in 5 wt.% composites were visible through SEM analysis, displayed in Fig. 2e. Even at a lower magnification of 3,000×, multiple sites of agglomerated GnPs were identified, leading to a non-uniform fracture surface with many areas devoid of nanoplatelets. A low magnification cross-section of the impact fracture surface for 3 wt.% loading is visible in Fig. 2f. Throughout the fracture surface, GnPs can be identified primarily perpendicular in orientation, indicating the majority of this filler is aligned in the direction of melt flow during processing. It is also observed that BioC distribution was relatively uniform throughout the sample surface. Graphene’s hydrophobicity is largely dependent on thickness, with thinner layers being more hydrophilic, which in turn increases liquid bridging forces between the graphene sheets^29^. There was no evidence through SEM analysis of interactions occurring between BioC particles and GnPs. This was to be expected given the highly carbonaceous nature of the fillers, and consistent with literature findings of fillers of this nature^30^.

**Figure 3:**
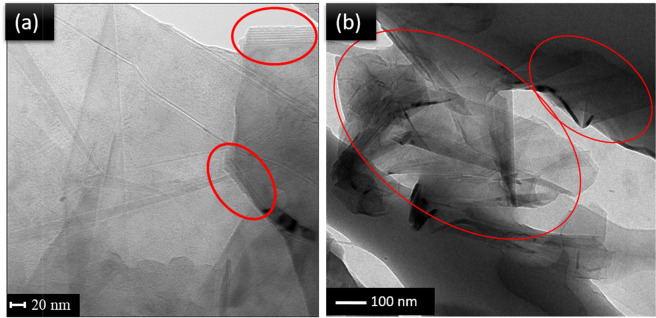
TEM image of PP/BioC/MA-g-PP/GnP (77/17/3/3) composite, with stacked GnP sheets identified at two different magnifications **(a,b)**.

The 77/17/3/3 composite was further analyzed through AFM imaging, as displayed in Fig. S3. Modulus mapping of the sample surface led to insight into the dispersion of GnPs within the PP matrix, as GnP is characterized by a high modulus. The Young’s modulus of neat PP used in this study was found to be around 2.0 GPa, while a study by Kardashev *et al*.^31^ found pine wood BioC to have an elastic modulus ranging between 4–9 GPa. While this value will differ for BioC from different feedstocks, it does not compare to the natural modulus of GnP, which can range from 100 GPa to several TPa once introduced into a matrix^21^. Thus, the white spots seen in Fig. S3 are the distribution of GnPs in the matrix, and areas of agglomeration can be seen. There were large sections of the sample surface that were devoid of GnPs, confirming suspicions that there was poor dispersion of the reinforcing filler. TEM imaging of 3 wt.% GnP composites, displayed in Fig. 3, confirmed the results seen through SEM imaging. Agglomeration of multiple GnP layers were identified and circled.

### Mechanical properties

**Figure 4:**
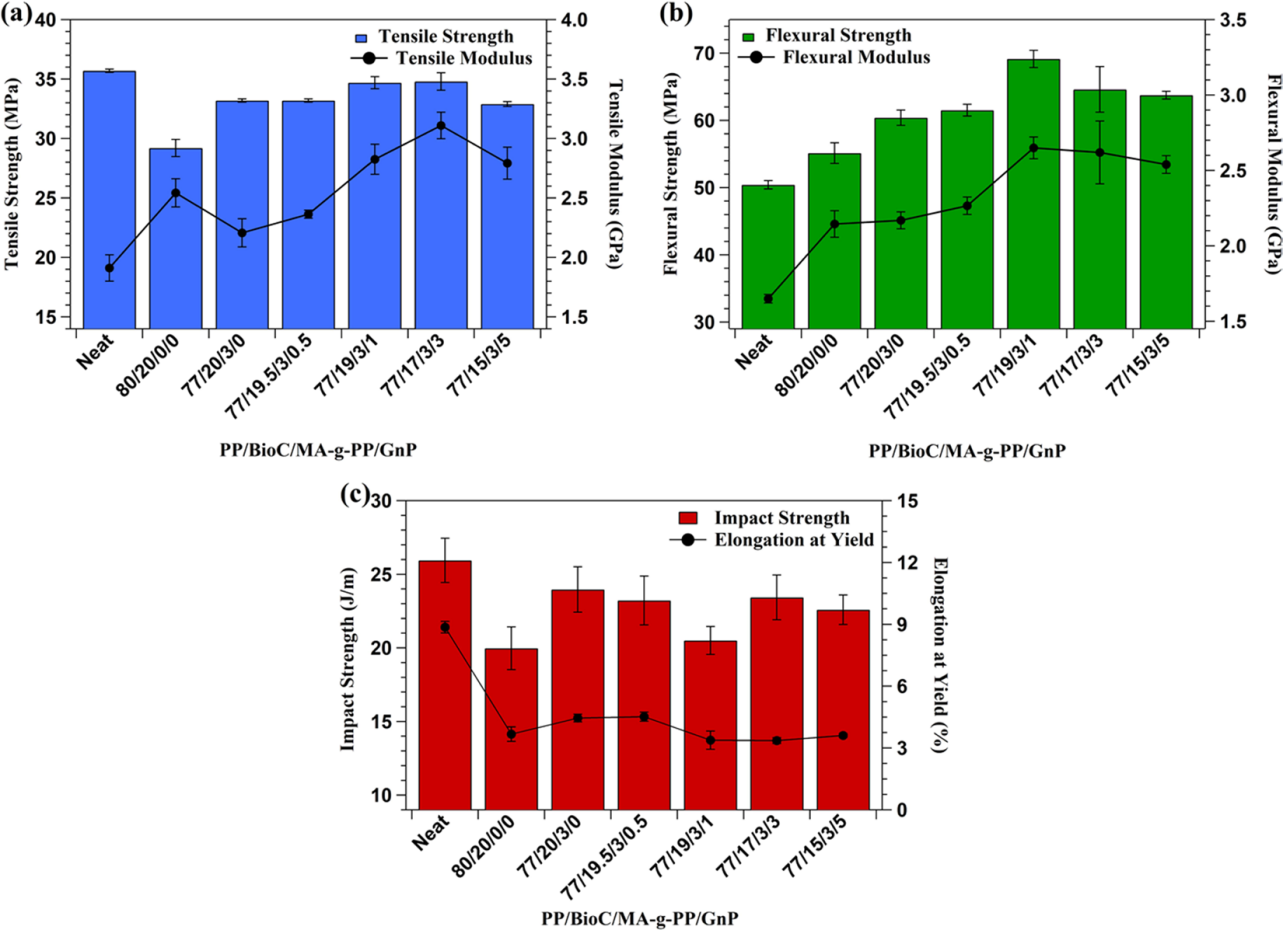
(**a**) Tensile strength and modulus, **(b)** flexural strength and modulus, **(c)** notched Izod impact strength and elongation at yield of neat PP and composites.

Figure 4 displays the tensile, flexural and impact strength of neat PP and all composites studied, as well as the moduli. Neat PP had a tensile strength around 35 MPa and a flexural strength around 50 MPa. It must be noted that neat PP and all composites were tested at different rates according to ASTM standards, making a direct comparison impossible. Typically, a faster strain rate correlates to greater tensile strength in composites. This was supported by a study that found neat PP3825 had a strength of ~27 MPa and modulus of ~1.4 GPA when tested at a rate of 5 mm/min^32^. It appeared that BioC had poor bonding with the PP matrix, rendering it less efficient in stress transfer, as corroborated by other findings^2,11^. Addition of MA-g-PP compatibilizer partly restored the tensile strength, enforcing the idea that improved adhesion and stress transfer between BioC and the PP matrix occurred, as suggested by SEM analysis. With 3 wt.% compatibilizer, tensile strength was raised from around 29 MPa to 33 MPa. Increasing GnP content led to minor improvements in tensile strength at 1 wt.% and 3 wt.% loadings, which then diminished again at 5 wt.%. The improvements seen are probably due to the large surface area GnPs offer for their comparatively small size, which acted to enhance stress transfer between matrix and filler^21^. M5 GnP is characterized by a particle thickness of 6–8 nm and a particle diameter of 5 µm, which is relatively small compared to many commercially available GnP grades. This is advantageous, as it has been found that reduced GnP size improves reinforcement properties^33^. However, the improvement in tensile strength seen is less pronounced than other findings due to poor dispersion of GnPs in the PP matrix. Tensile strength is thought to have decreased in the 5 wt.% GnP blend due to excessive agglomeration, as higher loading levels increased the amount of interaction between graphene sheets. Further, a study by Wang *et al*.^34^ indicated that sudden mechanical property drops are common when using GnPs due to the formation of continuous graphene networks, which may be the case for high loadings like 5 wt.%.

Tensile modulus saw a large increase through addition of BioC due to particle stiffness that acted to restrict PP matrix mobility. A study by Codou *et al*.^14^ confirmed that *Miscanthus* BioC increased the storage modulus of PP composites through DMA testing, indicating a restriction of molecular mobility at the interface and a resulting increase in tensile modulus. The modulus improvement is supported by the heat deflection temperature (HDT) results in section 3.6.2, as addition of BioC served to prevent polymer chain deformation under load. Addition of compatibilizer diminished the modulus to 2.2 GPa due to its rubbery nature and naturally low elastic modulus^35^. Furthermore, incorporation of GnP significantly improved modulus up to 3 wt.%, with a 41% increase compared to the compatibilized blend. This is largely due to GnP material strength, which served to improve interfacial strength and prevent deformation in the elastic region of the matrix^36^. However, this strength was limited with higher loading levels of GnPs due to reduced dispersion, explaining the decrease in modulus seen with 5 wt.% GnP content.

Flexural strength and modulus are shown in Fig. 4b. They followed similar trends as seen in the tensile data, with the only differences being that BioC improved flexural strength and addition of compatibilizer had no significant effect on modulus. Improvements to strength can be attributed to the stiffness of BioC particles and GnP sheets. The even dispersion of BioC particles further improved flexural strength, but agglomeration of GnPs at high loading levels led to diminishing strength at 5 wt.% loading. Improvement of flexural modulus with addition of MA-g-PP can be attributed to improved bonding, as this served to restrict matrix deformation in the elastic region. Furthermore, alignment of GnPs to follow the direction of flow during extrusion and injection could also serve to improve modulus up to 3 wt.% content, as a study by Idumah *et al*.^36^ suggested. This was observed through SEM imaging in both Fig. 2d,f. Past this loading level, agglomeration diminished both flexural strength and modulus. Furthermore, representative stress/strain curves for tensile data for the biocomposites tested can be found in Fig. S4.

Impact strength of neat PP and all biocomposites tested is shown in Fig. 4c. Neat PP had an impact strength around 26 J/m, and all composites tested had values within standard deviation after undergoing a complete break, except 1 wt.%, 5 wt.%, and the uncompatibilized blend. A slight decrease in impact strength was observed on addition of BioC due to the incompatibility with the PP matrix, but this was recovered through incorporation of compatibilizer. GnP addition had no observable effect on impact strength and, in the case of 1 and 5 wt.%, diminished it beyond the compatibilized blend. The benefit of GnPs on impact strength are also limited due to the increased density of the composites and heterogenous dispersion throughout the fracture surface, as observed in SEM imaging^36^. This imposes a trade-off between stiffness and toughness of the material, with one blend presenting an optimal balance. The effect of GnP on impact strength was supported by a recent study on PP/kenaf fibre/GnP composites^36^. Fig. 4c also displays the elongation at yield of neat PP and the biocomposites, which displays a sharp decrease upon BioC loading. Overall, the trends of elongation at yield followed well with the impact strength of the different formulations, as both depended on the material’s ability to deform under stress. At high strains, the material acted in a more brittle fashion and is another indication of poor compatibility between the BioC particles and the PP matrix^14^.

From the mechanical data collected in this study, it was apparent that both a 1 wt.% and 3 wt.% GnP loading level provided a similar stiffness-toughness balance. These blends maintained the impact strength of neat PP, while maximizing tensile strength and modulus, as well as improving flexural strength and modulus. Further examination of thermal properties was needed to elucidate an optimal blend.

### Physical analysis of composites

#### Melt flow index (MFI)

MFI is a relevant characteristic of composites as it describes ease of flow of the thermoplastic melt. It is also inversely proportional to the average molecular weight of a material, with higher MFI’s found to correlate with a lower molecular weight^37^. The MFI of neat PP and all composites tested is found in Table 2. The grade PP1350N was chosen due to its high MFI to allow it to achieve good flow characteristics, which in turn allowed for lower cylinder temperatures and a shorter cycle time. The addition of fillers leads to a subsequent decrease in MFI, making a neat polymer with low viscosity desirable. This could be attributed to a lower molecular weight, and further nucleation additives allow for decreased polymer cooling time. BioC particle size and aspect ratio resulted in lowering the MFI from 55.5 g/10 min to 19.1 g/10 min due to entanglement of PP polymeric chains. This served to increase the viscosity of the polymer, leading to a lower MFI^38^. These findings matched a similar study on the addition of switch grass BioC to a high-density polyethylene matrix^39^. MFI was slightly diminished on addition of compatibilizer, which indicated formation of good adhesion between BioC particles and the PP matrix. This hypothesis was reinforced by the results seen through SEM analysis. Addition of 0.5 and 1 wt.% GnP content raised MFI from the compatibilized PP/BioC blend, and this is attributed to the lubricating properties of GnPs. The unique multilayered structure served to reduce contact between sliding bodies, as found in similar studies^40–42^. However, at 3 and 5 wt.% GnP loading levels, MFI decreased. This was due to significant agglomeration, which reduced lubricating capability and increased viscosity due to the large aspect ratio of GnPs, and is consistent with previous studies^35,36,38^.

**Table 2:** MFI and density of neat PP and composites.

Sample (PP/BioC/MA-g-PP/GnP)	MFI (g/10 min)	Density (g/cm^3^)
Neat PP	55.48 ± 2.657	0.908 ± 0.004
80/20/0/0	19.10 ± 0.0453	0.985 ± 0.004
77/20/3/0	17.28 ± 0.059	0.988 ± 0.001
77/19.5/3/0.5	18.58 ± 0.161	0.984 ± 0.002
77/19/3/1	19.92 ± 0.13	0.984 ± 0.001
77/17/3/3	15.21 ± 0.02	0.989 ± 0.002
77/15/3/5	13.76 ± 0.10	0.994 ± 0.001

#### Density

Maintaining low density composites is essential for automotive applications, as any weight reduction serves to improve fuel efficiency. BioC powder has a density ranging from 1.3–1.4 g/cm^3^, while the GnPs used have a density of 2.2 g/cm^3^, which is much lower compared to common inorganic fillers, such as calcium carbonate that has a density of 2.83 g/cm^3^, or talc that has a density between 2.6–2.7 g/cm^3^ ^43^. PP had a density around 0.91 g/cm^3^, and addition of BioC increased this to 0.99 g/cm^3^, which remained relatively constant upon further addition of GnPs. As seen in Table 2, the density of PP and all biocomposites was below 1 g/cm^3^. In comparison, another study found that utilization of 20 wt.% talc filler in a PP matrix resulted in composites with a density around 1.058 g/cm^3^, denser than the PP/BioC/GnP formulations^43^. Thus, substitution of traditional mineral fillers with BioC has the potential for weight reduction of fabricated parts.

### Thermal characterization

#### Thermogravimetric analysis (TGA)

**Figure 5:**
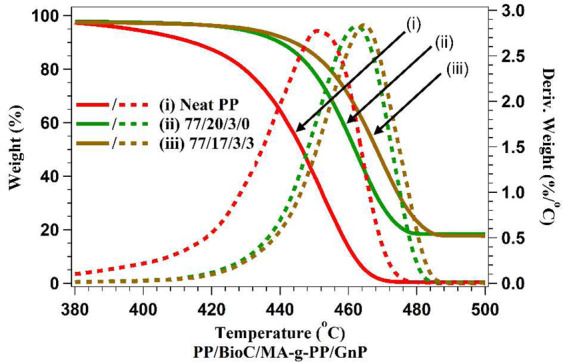
TGA (solid line) and derivative TGA curve (dashed line) of neat PP and composites.

Thermal stability of the composites was analyzed through TGA testing, an extremely important property for automotive parts to ensure proper function over time. The results obtained are displayed in Fig. 5 and Table 3. Figure 5 shows the change in weight as temperature was increased over time for neat PP, the compatibilized PP/BioC blend, and 3 wt.% GnP content. From this graph, the temperature at which 10 wt.% (*T*_10_) of the material had degraded and char yield were determined. Neat PP had a *T*_10_ of 419 °C, which increased to 438 °C for the compatibilized PP/BioC blend. GnP addition led to a minor increase, with 3 wt.% raising *T*_10_ to 444 °C, being the largest increase of all GnP blends tested. The rightward shift in Fig. 5 indicated that GnP inclusion resulted in more thermally stable composites, as chain scission and depolymerization occurred at higher temperatures^44^. BioC has strong thermal stability due to a loss of functionality during the pyrolysis process^7^. GnP also lacks functional groups, confirmed through FTIR testing, that improve its stability. This was seen through the difference in char yield between neat PP and the biocomposites. Neat PP only had 0.1% material remaining following TGA heating, while BioC and GnP had close to 17% char yield as the majority of the BioC and GnPs persisted. This trend was also seen in PP/polyamide 6/BioC composites in a study conducted by Codou *et al*.^14^

**Table 3:** TGA and DSC data of neat PP and composite samples.

Sample (PP/BioC/MA-g-PP/GnP)	TGA data		DSC data				
	*T*_10_ (°C)	*T*_*max*._ (°C)	First Heating		Second Heating		Cooling
			*T*_*m*_ (°C)	*X*_*c*_ (%)	*T*_*m*_ (°C)	*X*_*c*_ (%)	*T*_*c*_ (°C)
Neat PP	418.6 ± 5.9	450.9 ± 1.8	167.8 ± 0.3	41.9 ± 0.1	164.6 ± 0.6	50.9 ± 0.4	121.5 ± 0.1
80/20/0/0	442.2 ± 1.4	463.8 ± 2.6	167.6 ± 1.8	43.6 ± 0.2	165.0 ± 0.1	51.9 ± 0.1	127.4 ± 0.5
77/20/3/0	438.1 ± 2.7	461.5 ± 1.5	167.4 ± 1.0	44.6 ± 0.2	164.9 ± 0.1	51.9 ± 0.4	126.9 ± 0.2
77/19.5/3/0.5	438.0 ± 0.6	461.4 ± 1.0	168.0 ± 0.2	45.1 ± 0.5	165.0 ± 0.1	52.4 ± 0.1	127.5 ± 0.3
77/19/3/1	439.7 ± 0.69	464.8 ± 0.1	166.7 ± 0.9	45.0 ± 0.4	165.2 ± 0.3	52.8 ± 0.4	128.2 ± 0.2
77/17/3/3	444.1 ± 0.19	468.6 ± 0.5	167.3 ± 0.1	45.3 ± 0.1	165.8 ± 0.4	53.0 ± 0.4	129.0 ± 0.1
77/15/3/5	438.6 ± 0.47	463.1 ± 0.3	166.8 ± 0.2	44.5 ± 0.3	165.7 ± 0.2	53.0 ± 0.1	129.9 ± 0.3

(*) *T*_10_ and *T*_*max*_. are the decomposition temperatures at 10 wt.% loss and the first derivatives peak, respectively, as determined through TGA measurements. Melting (*T*_*m*_) and cooling (*T*_*c*_) temperatures, as well as degree of crystallinity (*X*_*c*_), were determined through DSC measurements of both the first and second heating scans.

The derivative weight of composites is also displayed in Fig. 5 and Table 3, with the peak corresponding to the temperature at which degradation occurred at a maximum rate. Neat PP had a maximum degradation temperature of 451 °C, which increased to 461 °C in the compatibilized PP/BioC blend, and then further increased to 469 °C on addition of 3 wt.% GnP. BioC particles dispersed in the PP matrix assisted with even heat distribution due to their thermal stability and resistance to degradation, as corroborated by a study of wood/PP/BioC composites conducted by Das *et al*.^28^ Further increase in thermal stability through addition of GnPs was likely due to graphene’s barrier properties and its ability to remove antioxidants, which protected PP from oxidative degradation^45^. The ability of GnPs to act as a thermal conductor and their high surface area also served to help improve heat spread along the polymer matrix, and it is hypothesized that improved dispersion would increase thermal stability further. The decrease in maximum degradation temperature at 5 wt.% loading supports this, as SEM analysis in Fig. 2e shows large agglomeration of the nanofiller with other areas devoid of GnPs. Furthermore, the effect of addition of both GnPs and BioC on flame retardance in PP composites have been analyzed elsewhere. It was found that both fillers serve to form barriers against heat transport through hindrance of O_2_ movement. At similar loading levels, the peak heat release rate saw a significant decrease from addition of either filler, and indicates that the biocomposites in this study should benefit from higher flammability retardation as well^46,47^. Supplementary data on the thermal and electrical conductivity of the composites is found in Figure S4, illustrating the minor augmentation observed in both conductivities on inclusion of GnPs in the formulations. The small diameter of GnPs used was beneficial, as a recent study found smaller GnP size led to improved thermal stability of composites^25^.

#### Differential scanning calorimetry (DSC)

**Figure 6:**
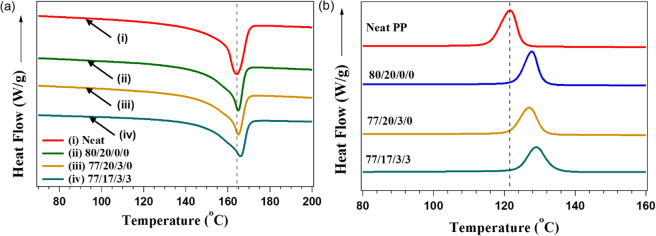
DSC of **(a)** second heating and **(b)** cooling curve of neat PP and composites.

The thermal characteristics were determined through DSC analysis, summarized in Fig. 6 and Table 3. Crystallinity was lower for all composites during the first heating ramp as previous thermal history had not been erased, resulting in a higher amorphous content compared to the second heating ramp. Crystallization temperature occurs at the peak during the cooling cycle, as seen in Fig. 6b. At this point, polymeric PP chains reorganized as the amorphous structure began crystallization. Neat PP had a *T*_c_ of 121.5 °C, which increased to 127.4 °C on addition of BioC. These results indicate that BioC acted as a nucleating agent for PP. Abdelwahab *et al*.^2^ showed that the incorporation of fillers such as talc, glass fiber and BioC increased the *T*_c_ of PP. This also indicated that use of BioC pyrolyzed at higher temperatures could prove more effective in raising *T*_c_, as this BioC has a higher ash content. To a lesser extent, incorporation of GnPs also raised *T*_c_, showing that they also served as nucleating agents. The morphology of GnPs gives a large surface area for crystallization to occur at relatively higher temperatures, and this trend had also been found in PP/GnP composites studied elsewhere^25^. However, the effect was less pronounced than addition of BioC, probably due to agglomeration of GnPs, as this would reduce the surface area available for crystallization to occur.

In both the first and second heating cycles, crystallinity had a minor increase with addition of BioC and the subsequent incorporation of GnPs. However, based on the filler content, this is rather insignificant, as in the second heating cycle neat PP crystallinity increased from 51% to around 53% in the 3 wt.% GnP composite. Likewise, melting temperature remained relatively invariant on addition of fillers and compatibilizer, as seen in Fig. 6a. The melting peak of neat PP was seen around 168 °C, which corresponds to the α-crystalline phase of PP^2^. As *T*_m_ is largely dependent on the forms of crystals present, these results indicate that neither BioC nor GnP significantly affected crystal type. GnPs have been seen to cause β-crystal formation in PP composites that are characterized by a secondary melting peak between 150–160 °C, but in insignificant amounts and as such do not affect T_m_^25^. The formulations tested in this study showed no sign of a secondary melting peak.

### Thermomechanical characterization of composites

#### Heat deflection temperature (HDT)

**Figure 7:**
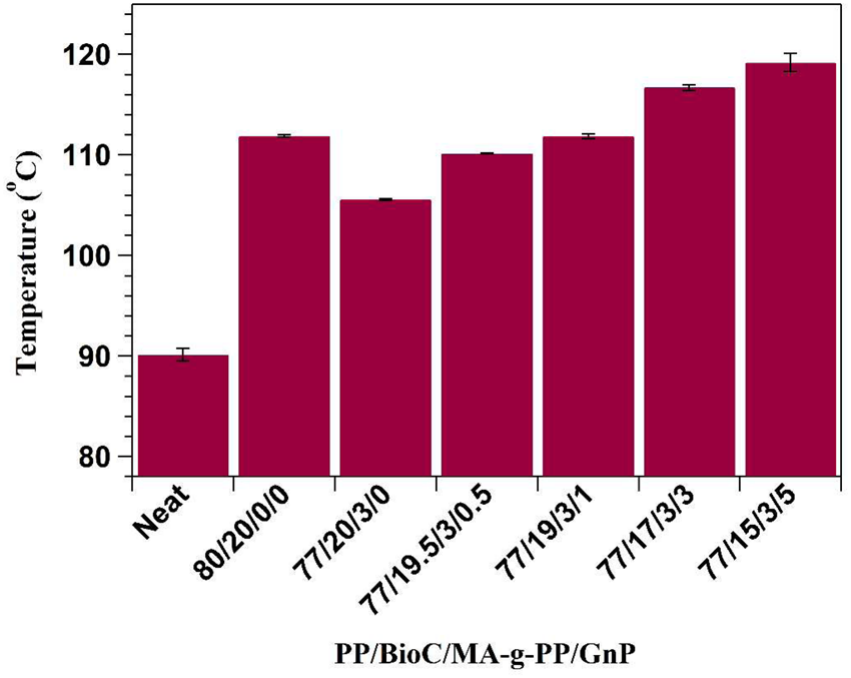
HDT of neat PP and composites.

HDT is another important material characteristic for automotive applications as it describes heat resistance to a specified load. HDT values of PP and all biocomposites are displayed in Fig. 7. The HDT of neat PP was 90 °C, which dramatically increased to 112 °C on inclusion of BioC. A similar result was found by a study looking at polyamide 6/BioC composites^12,15^. BioC particles restricted PP chain mobility, limiting material deformation when subjected to a force. Incorporation of compatibilizer served to lower HDT, as expected from its rubbery nature and the lowered tensile modulus of this formulation. However, once GnP was added to the compatibilized PP/BioC blend, increasing loading led to a relatively linear increase in HDT. GnP led to enhancement of modulus, explaining the subsequent increase in HDT, as this material also served to block chain mobility of PP. These HDT findings are supported by a similar study by Idumah *et al*.^36^

#### Coefficient of linear thermal expansion (CLTE)

**Figure 8:**
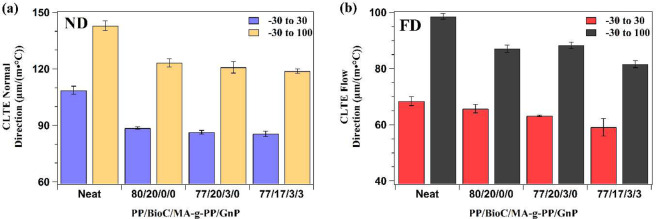
CLTE of neat PP and composites, measured both in **(a)** the normal direction (ND) and **(b)** the flow direction (FD).

CLTE describes the rate of change of material size with varying temperature and gives insight into the dimensional stability of the material. CLTE is typically evaluated in two distinct regions, the glassy and rubbery states. At lower temperatures in the glassy state, there is a lack of molecular motion, while motion of polymeric chains in the rubbery state is more frequent due to the higher temperature. As such, the rubbery state has a higher CLTE, since chain movement is less restricted and expansion is easier. The glassy state of PP and biocomposites was measured from −30 to 30 °C, and the rubbery state was analyzed from −30 to 100 °C, as per ASTM D696. Between these two states lies the glass transition temperature (*T*_*g*_), described as an area of polymer matrix relaxation and an increase in free volume. While not measured in this study, different forms of PP (isotactic, atactic, and syndiotactic) have a glass transition region ranging from −20 to 0 °C, but can vary up to ~15 °C depending on the grade^15^. A recent study by Abdelwahab *et al*.^2^ found that the glass transition for grade PP1350N was 15.7 °C according to tan δ curves obtained through dynamic mechanical analysis. Thus, to follow ASTM standards for PP, the data collected includes the glass transition and does not solely describe the glassy and rubbery states, but rather serves as a comparison in trends between formulations tested. Both normal direction (ND) and flow direction (FD) were tested in this study and results can be found in Fig. 8a,b, respectively.

Many polymers have a high CLTE, an undesirable property for automotive parts. Neat PP had a CLTE from −30 to 30 °C in the ND of 109 µm/(m· °C), which was lowered by 18.5% to 88.6 µm/(m· °C) upon addition of BioC. Addition of compatibilizer and GnP led to subsequent decreases, but only by 2.5% and 1%, respectively. A similar trend was also seen from −30 to 100 °C in the rubbery region, as well as both measurements in the FD. One noticeable difference in the FD was that addition of GnP led to a more significant decrease in CLTE. In the compatibilized PP/BioC blend, CLTE was diminished by 7.6% on addition of 3 wt.% GnP, a change from 88.3 µm/(m· °C) to 81.6 µm/(m· °C) as measured from −30 to 100 °C. This data suggests that BioC acted to constrain polymer movement due to its high available surface area and dispersion within the matrix, supported by other recent studies^2,14,15^. Addition of MA-g-PP compatibilizer further reduced CLTE through improvement of bonding between PP and BioC particles. Similarly, GnPs served to lower CLTE due to their high available surface area and low coefficient of thermal expansion^36^. The greater improvement in the FD suggested higher GnP alignment in the direction of polymer flow during the molding process, as this would expose a larger surface area to restrict chain movement.

## Conclusions

Compatibilized PP/BioC/GnP composites were prepared through injection molding, and the effect of varying GnP content was analyzed. Morphological analysis highlighted the presence of voids and a lack of adhesion in the PP/BioC composite, which was improved through addition of compatibilizer. There was evidence of significant agglomeration of GnPs in the compatibilized PP/BioC matrix due to adsorption of moisture and intermolecular forces between platelets, which was reinforced by mechanical and thermal data. Addition of compatibilizer and GnP improved tensile and flexural strength of the 20 wt.% BioC composites, as well as both moduli by ~41% and ~21%, respectively. However, GnP addition had no significant effect on impact strength. It was determined that 3 wt.% loading of GnP led to optimal properties, but the effect of this nanomaterial was limited due to agglomeration. BioC and GnPs both served to improve the thermal stability of the composites, as seen through TGA analysis, by ensuring even heat distribution. DSC testing showed that crystal type and overall crystallinity remained relatively constant on addition of both fillers, but they did serve to improve crystallization temperature, indicating that they were possible nucleating agents. The HDT of neat PP was significantly improved by incorporation of both BioC and GnPs due to restricted chain mobility, with the optimized blend having a 29.5% increase. The dimensional stability of composites was also analyzed, which both fillers improved by lowering CLTE through constraining polymer movement. Overall, these composites have the potential to benefit the automotive field, as almost all thermal and mechanical properties saw improvement beyond the neat polymer. Additionally, these formulations had a higher biocontent and lower density compared to mineral filled PP composites, thereby reducing dependence on petroleum products.

## Experimental

### Materials

The soyhull meal used was purchased locally from Nieuwland Feed & Supply Ltd. based in Elora (Ontario, Canada). The chemical composition of the soyhull meal consisted of 5% cellulose, 15% hemicellulose, 2% lignin, 44% acid detergent fiber, 10% crude protein, ash content below 5% and trace minerals. Acid detergent fiber is primarily comprised of cellulose, and to a lesser extent, lignin and silica. Thus, the true cellulose content is likely closer to the average 20% for soybean hulls^48^.

Polypropylene (PP), grade PP1350N, was purchased from Pinnacle Polymers, USA. It was characterized by an MFI of 55 g/10 min, notched Izod impact strength of 25.9 J/m, density of 0.9 g/cm^3^, and elongation at yield of 9%, which coincides well with the technical data sheet. The MA-g-PP compatibilizer, under the commercial grade Fusabond P353, was acquired from Dupont (NC, USA). It had a density of 0.904 g/cm^3^ and MFI of 22.4 g/10 min, as described by the technical data sheet. The nano-filler used in this work was M5 GnPs supplied by XG Sciences (Lansing, USA), characterized by a surface area of 120 to 150 m^2^/g, thickness between 6 to 8 nm and average particle diameter of 5 µm, as indicated on the technical data sheet. Its structure had short stacks of graphene sheets that came together to form a platelet shape.

### BioC preparation

The soyhull meal was pretreated by drying at 80 °C for a minimum of 24 h prior to pyrolysis until a moisture content below 2 wt.% was achieved, as measured by a moisture analyzer. Pyrolysis of the fibers was conducted in a Carbolite GLO 10/11–1 G industrial front loading retort furnace with a horizontal configuration. Approximately 1 kg of soyhull meal was put in a steel vessel and then placed in the furnace. Once closed, the furnace atmosphere was flushed with nitrogen gas to create an inert atmosphere. A heating rate of 7.5 °C·min^−1^ was employed to reach the set operating temperature of 500 °C. This temperature was maintained for a residence time of 60 minutes, at which point the furnace was cooled to room temperature prior to removal of the sample. The low temperature BioC was then collected and ball milled using a Pulverisette 5 (Fritsch GmbH Idar-Oberstein, Germany), for two hours in alternating 30 minute forward and reverse rotation cycles. This process was performed with 354 g of zirconium oxide balls and 65 g of BioC per chamber, with a total of 4 chambers. The samples were collected following ball milling and left to cool for a minimum of one day. The BioC did not undergo sieving prior to incorporation into composites.

### Biocomposites fabrication

Prior to composite fabrication, all materials except the hydrophobic PP pellets were dried at 85 °C for a minimum of 24 hours to prevent moisture absorption that could lead to hydrolysis reactions. Composites were prepared in the proportions indicated in Table 4. To improve surface adhesion of BioC and PP, the compatibilizer MA-g-PP at 3 wt.% was employed. Flexural, tensile and impact samples for each formulation were prepared through melt processing in a Xplore MC 15 Micro-compounder (Netherlands) at 180 °C, a screw speed of 100 rpm and a residence time of 2 minutes, followed by moulding in a 12cc micro injection moulding machine at 30 °C and 10 bar for 20 s.

**Table 4:** Sample designations and component proportions of the composites.

Sample (PP/BioC/MA-g-PP/GnP)	PP (Wt.%)	BioC (Wt.%)	MA-g-PP (Wt.%)	GnP (Wt.%)
Neat PP	100	0	0	0
80/20/0/0	80	20	0	0
77/20/3/0	77	20	3	0
77/19.5/3/0.5	77	19.5	3	0.5
77/19/3/1	77	19	3	1
77/17/3/3	77	17	3	3
77/15/3/5	77	15	3	5

### Mechanical analysis

Flexural and tensile tests for the composites were carried out on a Universal Testing instrument, Instron Model 3382 (USA). Tensile properties of neat PP were measured following ASTM D638 with type 4 specimens at a rate of 50 mm/min, while all the composites were tested at 5 mm/min in order to conform to the ASTM standard. This standard dictated that the sample must break between 30 seconds and 5 minutes during the testing process. All samples underwent flexural testing following ASTM D790 at a crosshead speed of 14 mm/min. Impact samples were notched directly after processing using a notching cutter (TMI, USA) and left for 48 hours, as required by ASTM D256. Notched Izod impact strength was measured by a Monitor impact tester, HIT25P Plus (Zwick/Roell) with a hammer pendulum of 2.75 J. All mechanical data used 5 samples for each measurement.

### Scanning electron microscopy (SEM) analysis

Prior to imaging, flexural bars were placed in liquid nitrogen for 5 minutes and then fractured using a Monitor impact tester. The cryo-fractured biocomposites were analyzed using a Phenom ProX (Netherlands) microscope. Accelerating voltage of 15 kV was applied for all composite imaging. The impact fracture surface was sputter coated with gold for 10 seconds prior to imaging.

### Atomic force microscopy (AFM) analysis

Prior to AFM imaging, injection moulded samples were microtomed through use of a Leica ultramicrotome to produce a flat sample surface. Measurements were performed on a Bruker AFM instrument Multimode 8 (Billerica, USA) utilizing the peak force quantitative nano-mechanical mode. Analysis of imaging was performed using Nanoscope software.

### Transmission electron microscopy (TEM)

GnP nanoparticles were examined through use of a Jeol 2010F microscope operated with an accelerating voltage of 200 kV. Prior to analysis, composite samples were prepared through collection of thin fragments from use of the Leica ultramicrotome. To allow tunneling to occur, sample width aimed to be below 100 nm during preparation.

### Fourier transform infrared spectroscopy (FTIR)

A Nicolet 6700 ATR-FTIR (Thermo Scientific, USA) was used to collect the spectra of powder samples and analyzed using OMNIC spectra software analysis. The spectra were collected using 64 scans per sample with a resolution of 4 cm^−1^.

### TGA-FTIR

A TA 5500 thermogravimetric analyzer system coupled with a Thermo Scientfic Nicolet iS20 Mid-Infrared FT-IR spectrometer was employed to conduct TGA-FTIR analysis. The chamber was put under a nitrogen atmosphere, set at a flow rate of 50 ml/min. Roughly 10 mg of soyhull fiber was heated at a rate of 20 °C/min from room temperature to 900 °C.

### Thermal analysis

Determination of melt temperature (*T*_m_), crystallization temperature (*T*_c_) and crystallinity (*X*_c_) of neat PP and the biocomposites was carried out with a DSC Q200 analyser, TA Instruments. Between 7–12 mg of specimen was loaded into a 40 µL aluminum pan and introduced into nitrogen gas at a flow rate of 50 mL/min. A heat/cool/heat cycle was employed, where the sample was equilibrated at −60 °C and heated at 10 °C/min to 210 °C, at which point it was cooled back down to −60 °C at a rate 10 °C/min and then reheated to 210 °C at the same rate. For each measurement, two samples were tested. Crystallinity was calculated using [Eq. (1)]

1 $${X}_{c}=\frac{\Delta {H}_{m}}{(1-\varnothing )\Delta {{H}_{m}}^{\ast }}\ast 100$$

where Δ*H*_*m*_ is the measured melting enthalpy (J/g) in the DSC experiment, calculated as the integral of the fusion peak, *ϕ* is the weight fraction of reinforcing agent in the composite, and *ΔH*_m_^*^ is the theoretical melting enthalpy of 100% crystalline PP (207.1 J/g)^2^.

Thermogravimetric analysis was performed on a TGA Q500 analyzer, TA Instruments. Between 10–15 mg of sample was added to a platinum pan and heated to 850 °C at a rate of 10 °C/min under a nitrogen flow of 60 mL/min. Two specimens were tested for every measurement.

### Thermomechanical analysis

Heat deflection temperature (HDT) tests were performed on a Dynamic Mechanical Analyzer (DMA) Q800, TA Instruments. ASTM D648 was followed for all testing, with a load of 0.455 MPa and a heating rate of 2 °C/min using a three-point bending cantilever. Two samples were tested for each HDT measurement.

Determination of the coefficient of linear thermal expansion (CLTE) was performed using a ramp rate of 5 °C/min and a force of 0.05 N on a Thermomechanical Analyzer (TMA) Q400, TA Instruments. The sample was first heated to 80 °C starting from −60 °C, and then cooled back down to −60 °C. The displacement change was then recorded as the sample was heated to 130 °C. Both the normal and flow directions were tested using expansion mode, with specimen dimensions of 3.2 mm/6.0 mm/6.0 mm. A nitrogen flow rate of 50 mL/min was employed, and two specimens were tested for every measurement. CLTE was calculated according to [Eq. (2)]:

2 $$\alpha =\varDelta L/{L}_{o}\varDelta T$$

where *α* is the coefficient of linear thermal expansion, *ΔL* is the change in length due to heating, *L*_*o*_ is the specimen length at room temperature, and *ΔT* is the temperature change tested in the experiment ( °C).

### Melt flow index (MFI)

The MFI of neat PP and all biocomposites was conducted on a Melt Flow Indexer (Model 2000A, Qualitest) following ASTM D1238. Tests were conducted at 230 °C using a load of 2.16 kg.

### Density

Densities of all specimens were performed on a MD-300S Densimeter (Alfa Mirage, Japan). As the samples floated in water, the Archimedes principle was utilized to determine density by using a sinker to keep samples under water during measurements.

### Thermal conductivity

A Hot-Disk TPS 500 Thermal Constants Analyzer from ThermTest, Inc. (Canada) was used to measure thermal conductivity, based on the transient plane source method with a Kapton disk sensor. Test parameters included a heating power of 120 mW and a measurement time of 40 s and is present in Fig. S5a.

### Electrical conductivity

The electrical conductivity of the sample formulations was measured at room temperature with a two-probe method in the normal direction with an Autolab PGSTAT302N (Metrohm Autolab B.V, Netherlands). Nova 1.8.17 software was used to measure the current over a potential range from −5 to 5 V, followed by determination of the slope from the IV curve. The IV curve was linear but contained significant variable signal-to-noise due to the low conductivity of the samples. As such, conductance was calculated based on Ohm’s law and converted to conductivity according to sample dimensions to allow for easy interpretation. This data can be seen in Fig. S5b.

## Supplementary information

Supplementary Information.
