# Supplementary material for: Hybrid biocomposites from polypropylene, sustainable biocarbon and graphene nanoplatelets
Source: Sci Rep. 2020 Jul 1;10:10714. doi: 10.1038/s41598-020-66855-4 (PMC7329909; doi:10.1038/s41598-020-66855-4)
Supplement: Supplementary file 1 — Supplementary Information. [file 41598_2020_66855_MOESM1_ESM.docx]

**Hybrid biocomposites from polypropylene, sustainable biocarbon and graphene nanoplatelets**

Ethan Watt^1^, Mohamed Abdelwahab^1,$^, Michael Snowdon^1,2^, Amar K. Mohanty^1,2*^, Hamdy Khalil^3^, Manjusri Misra^1,2*^

*^1^Bioproducts Discovery and Development Centre, Department of Plant Agriculture, Crop Science Building, University of Guelph, Guelph, N1G 2W1, Ontario, Canada*

*^2^School of Engineering, Thornbrough Building, University of Guelph, Guelph, N1G 2W1, Ontario, Canada*

*^3^ Woodbridge Foam Corporation, 4240 Sherwoodtowne Boulevard, Mississauga, Ontario L4Z 2G6 Canada*

*^*^Corresponding Author: Email:* [mohanty@uoguelph.ca](mailto:mohanty@uoguelph.ca) (A.K. Mohanty); mmisra@uoguelph.ca (M. Misra)

*^$^*Mohamed Abdelwahab is on leave from *Department of Chemistry, Tanta University, Tanta, 31527, Egypt*

**Supporting Information**

**
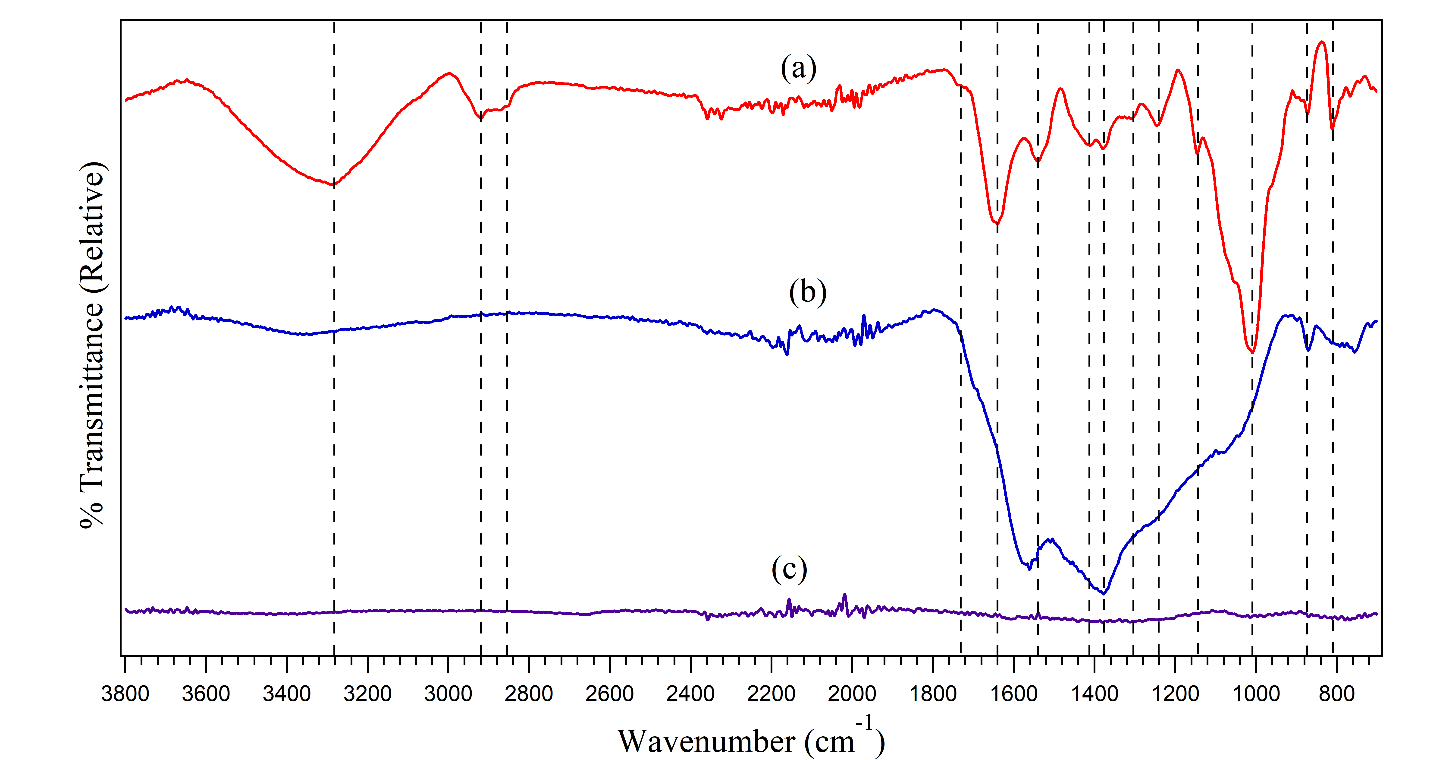
**

*Fig. S1. FTIR spectra of* ***(a)*** *raw soyhull meal,* ***(b)*** *soyhull BioC ball milled for 2 hours, and* ***(c)*** *M5 GnPs.*


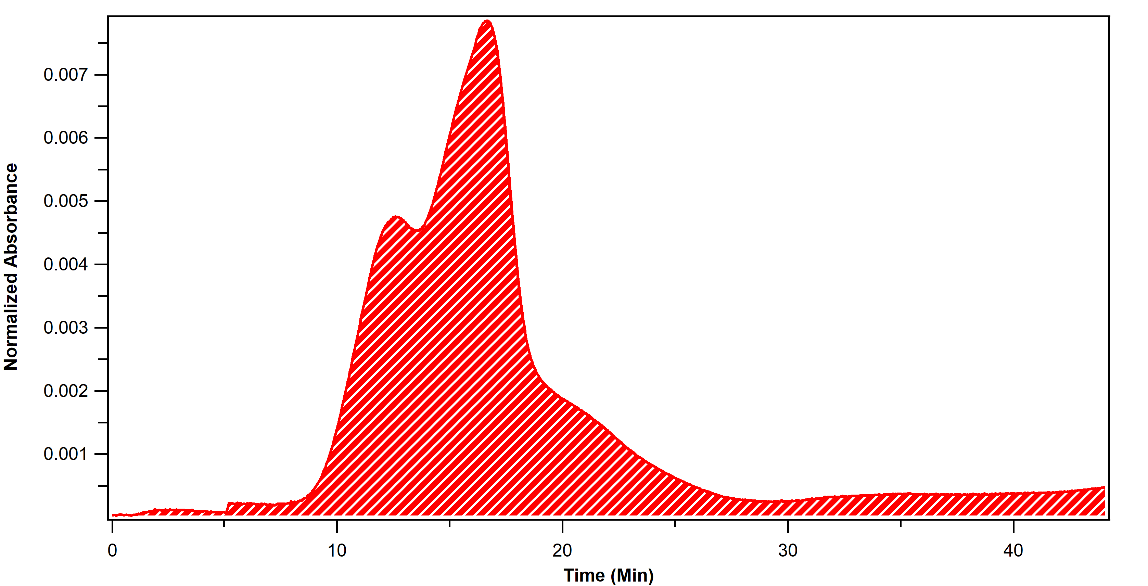


*Fig. S2. Normalized Gram-Schmidt curve of soyhull fiber heated over time.*

*
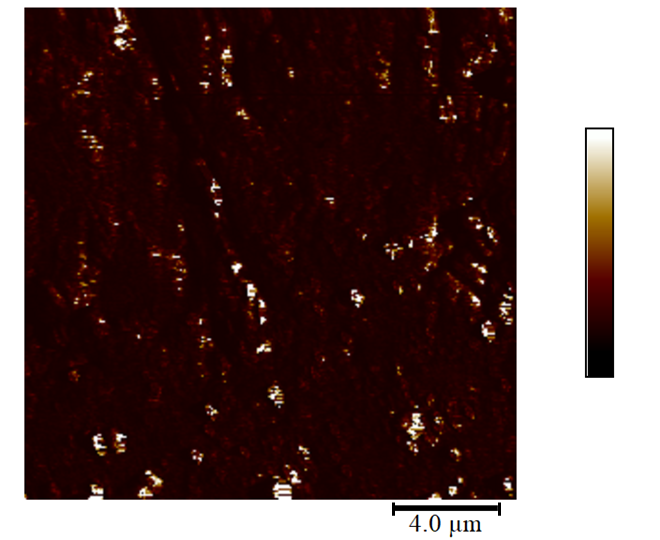
*

*Fig. S3. AFM modulus mapping for the PP/BioC/MA-g-PP/GnP (77/17/3/3) blend.*


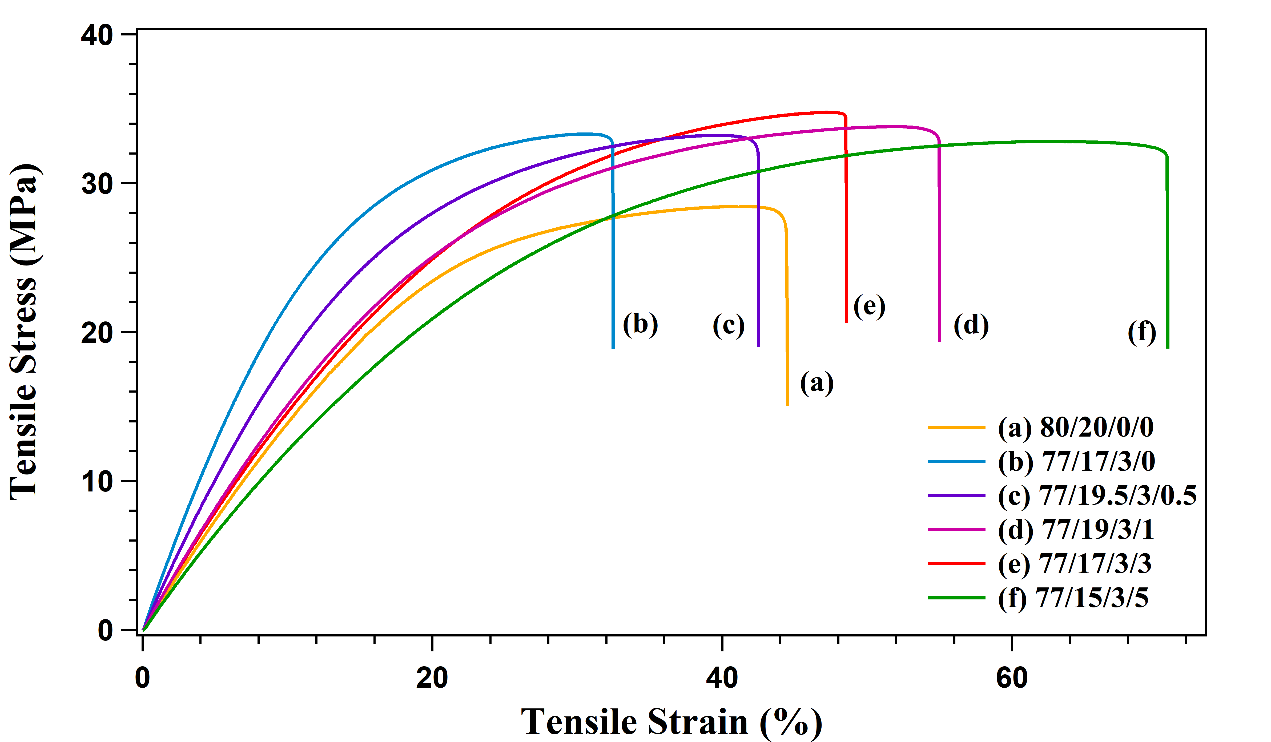


*Fig. S4. Tensile stress-strain curves of the biocomposites tested.*

*
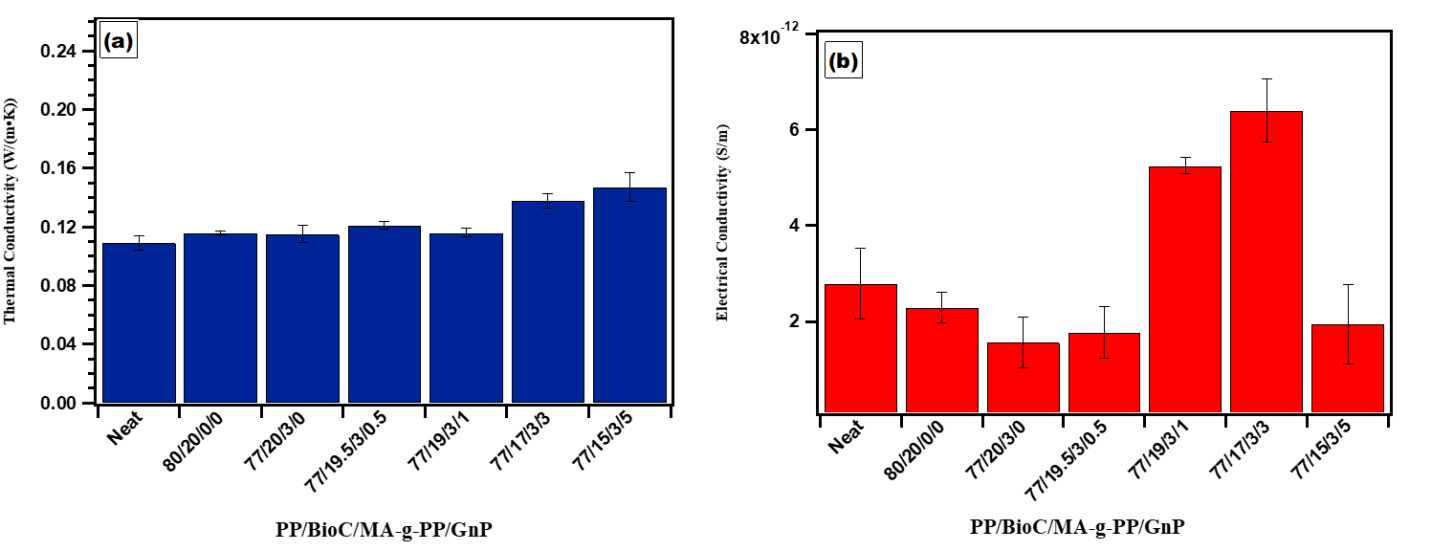
*

*Fig. S5: a) Thermal conductivity and b) Electrical conductivity of neat PP and composites*
